# The Asparaginyl Endopeptidase Legumain Is Essential for Functional Recovery after Spinal Cord Injury in Adult Zebrafish

**DOI:** 10.1371/journal.pone.0095098

**Published:** 2014-04-18

**Authors:** Liping Ma, Yan-Qin Shen, Harsh P. Khatri, Melitta Schachner

**Affiliations:** 1 W. M. Keck Center for Collaborative Neuroscience and Department of Cell Biology and Neuroscience, Rutgers University, Piscataway, New Jersey, United States of America; 2 Center for Neuroscience, Shantou University Medical College, Shantou, Guangdong Province, People's Republic of China; 3 Department of Basic Medicine, Jiangnan University Medical School, Wuxi, Jiangsu Province, People's Republic of China; University of Michigan, United States of America

## Abstract

Unlike mammals, adult zebrafish are capable of regenerating severed axons and regaining locomotor function after spinal cord injury. A key factor for this regenerative capacity is the innate ability of neurons to re-express growth-associated genes and regrow their axons after injury in a permissive environment. By microarray analysis, we have previously shown that the expression of *legumain* (also known as asparaginyl endopeptidase) is upregulated after complete transection of the spinal cord. *In situ* hybridization showed upregulation of *legumain* expression in neurons of regenerative nuclei during the phase of axon regrowth/sprouting after spinal cord injury. Upregulation of Legumain protein expression was confirmed by immunohistochemistry. Interestingly, upregulation of *legumain* expression was also observed in macrophages/microglia and neurons in the spinal cord caudal to the lesion site after injury. The role of legumain in locomotor function after spinal cord injury was tested by reducing Legumain expression by application of anti-sense morpholino oligonucleotides. Using two independent anti-sense morpholinos, locomotor recovery and axonal regrowth were impaired when compared with a standard control morpholino. We conclude that upregulation of legumain expression after spinal cord injury in the adult zebrafish is an essential component of the capacity of injured neurons to regrow their axons. Another feature contributing to functional recovery implicates upregulation of legumain expression in the spinal cord caudal to the injury site. In conclusion, we established for the first time a function for an unusual protease, the asparaginyl endopeptidase, in the nervous system. This study is also the first to demonstrate the importance of legumain for repair of an injured adult central nervous system of a spontaneously regenerating vertebrate and is expected to yield insights into its potential in nervous system regeneration in mammals.

## Introduction

In adult mammals, spinal cord injury (SCI) most often causes permanent disabilities due to failure to regenerate. In contrast to mammals, adult zebrafish regenerate successfully after SCI. Features leading to successful regeneration are the innate ability of neurons to re-express growth-associated genes, regrow their axons and adjust their synaptic connections in a permissive CNS tissue environment [1]. Thus, zebrafish have developed into a powerful model to elucidate the molecular mechanisms underlying not only spinal cord regeneration, but also regeneration of the adult CNS in general, raising the hope that the findings from zebrafish may lead to therapeutic approaches in mammals.

To identify novel regeneration-conducive molecules, we have performed mRNA microarray expression profiling of the nucleus of the medial longitudinal fascicle (NMLF), a brainstem nucleus containing neurons capable of axonal regeneration after injury, hypothesizing that genes that are upregulated in expression after SCI contribute to successful recovery of locomotor functions. One of the molecules upregulated in neurons capable of axonal regeneration after SCI was legumain [2], the function of which in regeneration and in nervous system functions in general, is unknown.

Since proteases play important roles in all aspects of nervous system development, tissue remodeling during learning/memory and after injury [3]–[4], we chose to investigate the unusual proteolytic enzyme legumain among the upregulated molecules. As a member of the C13 family of cysteine proteases, legumain/asparaginyl endopeptidase cleaves protein substrates at the C-terminus of asparagine [5]. Legumain was first observed to be located in the endosome/lysosome systems [6], has since been detected in the nucleus [7]–[8], at the cell surface [9] and in the extracellular matrix [10]–[13]. Legumain is involved in many physiological and pathological processes, such as antigen processing [14], cell migration [9] and proliferation [7], regulation of biosynthesis of lysosomal proteins [15], extracellular matrix turnover [12], as well as osteoclast formation and bone resorption [10]. Upregulation of legumain expression has been reported in various solid tumors, positively correlating with their invasive and metastatic potential [9], [16]–[17]. Legumain also functions as a carboxypeptidase [18].

The role of legumain in nervous system function has yet to be determined, particularly in recovery after injury. Here we report a novel function of legumain in the nervous system, and in particular in regeneration of the adult zebrafish CNS. Legumain expression is upregulated after SCI not only in regenerative brainstem neurons, but also in the spinal cord caudal to the lesion site. Inhibition of this expression reduces locomotor recovery, thus identifying legumain as a novel protease that is an important contributor to functional recovery after injury in the adult zebrafish CNS.

## Materials and Methods

### Spinal cord injury in adult zebrafish

Adult zebrafish (*Danio rerio*, male, age >6 months) were obtained from Aquatica Tropicals Inc. (Plant City, FL, USA). The fish were maintained at 28°C on a 14-h light and 10-h dark cycle. SCI was performed as described [2], [19]–[23]. Briefly, fish were put in phosphate-buffered saline (PBS, pH 7.4) containing 0.033% aminobenzoic acid ethylmethylester (MS222; Sigma, St Louis, MO, USA) for 5 min. To expose the vertebral column, a longitudinal incision at the left side of the fish was made. Then, a complete cut of the spinal cord was performed between the eighth and ninth vertebrae, 3.5 mm caudal to the brainstem-spinal cord junction. The sham-injured control fish (CON) received similar surgical procedures without cutting the spinal cord. The incision was sealed with Histoacryl (B. Braun, Melsungen, Germany) and fish were returned to their rearing tank. Fish were killed by an overdose of MS222 at the appropriate time points. All animal experiments were approved by the Rutgers University Institutional Animal Use and Care Committee (permit number: 10010), which conforms to NIH guidelines.

### Quantitative real-time polymerase chain reaction and microarray data

Four millimeters of whole spinal cord tissue caudal to the lesion site was collected at 1 day, 3 days, and 11 days after surgery from SCI or sham-injured fish. Spinal cords from 6 fish were pooled for each group. Total RNA was extracted using Qiagen RNeasy Micro Kit (Qiagen, Hilden, Germany) and a total of 200 ng RNA was used for first-strand cDNA synthesis using Superscript™ II reverse transcriptase (Invitrogen, Carlsbad, CA, USA) according to the manufacturer's instructions. Quantitative real-time polymerase chain reaction (qPCR) was carried out with Power SYBR Green PCR Master Mix (Applied Biosystems, Foster City, CA, USA) and the comparative cycle threshold Ct method (ΔΔCt method) was applied for data analysis as described [24]. No amplified product was observed when the cDNA template was replaced either by RNA sample without reverse transcription or by water. Results are expressed relative to sham-injured fish 1 day after SCI. The primers used were as follows: zebrafish legumain (forward: 5′-GGCGTTCCAGGGTAGCTCTA-3′; reverse: 5′-GACACTGGCTCCACTGCCTT-3′) and zebrafish ribosomal protein P0 (forward: 5′-TCGGCTACCCAACTCTTGCT-3′; reverse: 5′-TGTTTCGACAGTGACAGCCAG-3′).

Microarray analysis was performed and analyzed as described [2]. Please refer to previous publication for detailed information [2]. Data files for this microarray analysis have been deposited in the NIH Gene Expression Omnibus repository. The accession number is GSE28470 and the link is https://www.ncbi.nlm.nih.gov/projects/geo/query/acc.cgi?acc=GSE28470.

### *In situ* hybridization

PCR product for zebrafish *legumain* (NM_214759, 628–1043 bp of coding sequence) was cloned into pGEM-T Easy vector, with which digoxigenin-labeled RNA sense and antisense probes were transcribed *in vitro* using the Megascript™ system (Ambion, Austin, TX, USA) according to the manufacturer's protocol. In situ hybridization was carried out as described [2], [25]–[26]. Briefly, 25-µm-thick coronal brain sections or 20-µm-thick sagittal spinal cord sections were incubated with 0.1 N HCl for 10 min. After three washes in PBS, pH 7.4, the sections were treated with 10 µg/mL proteinase-K (Roche, Indianapolis, IN, USA) for 10 min at room temperature. Then, the sections were hybridized with sense or antisense probes at 55°C overnight. Alkaline phosphatase-coupled anti-digoxigenin antibody (Roche) was used to label the hybridized probes and the signal was developed with NBT/BCIP (nitro-blue tetrazolium and 5-bromo-4-chloro-3-indolyl phosphate, Roche). The same procedure was used for sections from fish that were not injured, sham-injured or spinal cord-injured. In situ hybridization with the sense control probe and the antisense probe was performed in parallel. No significant signal was observed for the sense probe. The NMLF (medial longitudinal fascicle) and IMRF (intermediate reticular formation) can be located according to the atlas of zebrafish brain [27] and the neurons with large cell body size (13–23 µm diameter) [20] are distinguishable from small glial cells [25]. Total positively stained neurons in the NMLF or the IMRF from each fish were counted. For profile measurements of spinal cord sections, cells in fifteen 10× fields, 5 from each animal, were counted. The intensity of each cell, in a total of 60 cells for each treatment, was measured using ImageJ.

### Immunohistochemistry and double staining for *in situ* hybridization and immunohistochemistry

Sections from brain (25-µm-thick coronal) or spinal cord (20-µm-thick, sagittal, 0–4 mm caudal to lesion site) were blocked with 1% bovine serum albumin and 3% donkey serum in PBS containing 0.2% Triton-X-100 for 1 hour at room temperature, followed by primary antibody incubation. Primary antibodies used were: goat anti-human legumain (AF2199, 1∶200, R&D Systems, Minneapolis, MN, USA), rabbit anti-glial fibrillary acidic protein (GFAP) (1∶500, Dako, Carpinteria, CA, USA), mouse anti-NeuN (1∶150, A-60, Millipore, Billerica, MA, USA), and mouse 4C4 antibody (92092321, 7.4.C4, 1∶60, Health Protection Agency Culture Collection). Legumain protein contains 433 amino acids, and the legumain antibody used here was raised against the human Legumain sequence of amino acids 18–433. The identity of these amino acids between human and zebrafish is 67%. The specificity of the legumain antibody in recognizing zebrafish Legumain was verified by Western blot analysis (Figure S1). Alexa Fluor secondary antibodies (Molecular Probes) were used at a dilution of 1∶600. Non-immune mouse or goat IgGs were used in place of primary antibodies at the same concentrations as isotype controls.

The immunohistochemical detection of the expression of NeuN (neuronal marker neuronal nuclei) in *legumain* mRNA positive cells (*in situ* hybridization) was carried out after completion of *in situ* hybridization. After three washes in PBS, the sections were treated with 10 mM citrate buffer (pH 6.0) at 95°C for 15 min for antigen retrieval as described [2], [28]. After cool down to room temperature, the sections were washed three times with PBS and blocked with PBS containing 1% bovine serum albumin and 3% goat serum. Then, the sections were incubated with mouse anti-NeuN antibody at 4°C overnight, followed by incubation with secondary antibody Alexa Fluor 555 (1∶600, Invitrogen).

### Application of morpholinos and biocytin

Two non-overlapping antisense morpholinos (MOs) for zebrafish *legumain* (NM_214759) (*legumain* MO1: 5′-GGCTCATTTCTGCAATTTACAGCTA -3′; *legumain* MO2: 5′-GTACACGACGCCCGAGCTGCCTGTA -3′, Gene Tools, LLC, Philomath, OR, USA) were used in this study. Both *legumain* MOs, tagged with carboxyfluorescein at the 3′ end, were designed to block translation. The specificity and efficacy of these two *legumain* MOs to knockdown Legumain expression was demonstrated by immunohistochemistry using an antibody against Legumain. The standard control MO (5′- CCTCTTACCTCAGTTACAATTTATA-3′) was also tagged with carboxyfluorescein at the 3′ end. MOs were prepared in Danieau solution (58 mM NaCl, 0.7 mM KCl, 0.4 mM MgSO_4_, 0.6 mM Ca(NO_3_)_2_, 5 mM HEPES, pH 7.6, as described [2], [19], [21]–[23].

Five hundred nanograms MO (approximately 0.2 µL, carried by Gelfoam (Upjohn, Kalamazoo, MI, USA)) was applied at the lesion site immediately after transection. Then, the fish were allowed to survive up to 6 weeks. The efficiency of this MO dose (500 ng per fish) was shown in our publication [19] and was confirmed thereafter [2], [21]–[23], [29]–[31]. In order to detect the neurons with regenerated axons, at 6 weeks after the MO treatment, biocytin (Sigma) (50 mg/mL, approximately 0.2 µL, absorbed in Gelfoam) was applied to a secondary lesion site, which was 3.5 mm caudal to the first spinal lesion site for MO treatment, i.e. 7 mm caudal to the brainstem–spinal cord junction. One day afterwards, the brains were dissected and biocytin was detected with the Vectastain ABC-DAB kit (Vector Laboratories, Burlingame, CA, USA) as described [2], [19], [22]. For cell body profile counting, all positively stained neurons in the NMLF and IMRF of each animal were counted.

### Locomotor analysis

To examine locomotor recovery, the total distance swum by the MO-treated fish was measured at 6 weeks after MO treatment. Freely moving fish was tracked as described [2], [19], [21]–[23]. Briefly, each fish was placed in a glass tank (50×30 cm) containing aquarium water (5 cm deep). The moving of the fish was recorded for 5 min by a camera mounted above the tank. Ethovision XT software (Noldus, Wageningen, The Netherlands) was used to track and calculate swim paths.

### Statistical analysis

A two-tailed Student's *t*-test was used to evaluate the results from microarray analysis of *legumain* mRNA expression in spinal cord, *legumain*-positive neuronal profiles in different regenerative nuclei after SCI, legumain immunostaining intensity in small cells, and numbers of legumain-immunopositive small cells after SCI. One-way ANOVA followed by Tukey's *post hoc* test, when appropriate, was used for analysis of Legumain protein expression profiles after MO treatment, locomotor recovery after MO application, evaluation of fluorescein-positive cell profiles after MO application and of neuronal profiles retrogradely labeled after MO application in two nuclei with innate capacity for regeneration. Two-way ANOVA followed by Tukey's *post hoc* test, when appropriate, was used for evaluation of the qPCR results of *legumain* expression in the caudal part of the spinal cord. The level of significance was set at *P*<0.05 for all analyses. Data are shown as mean values ±SEM. Statistical analyses were performed using R2.12.2 software (http://www.r-project.org).

## Results

### Legumain expression is upregulated in the nucleus of the medial longitudinal fascicle after SCI

**Figure 1:**
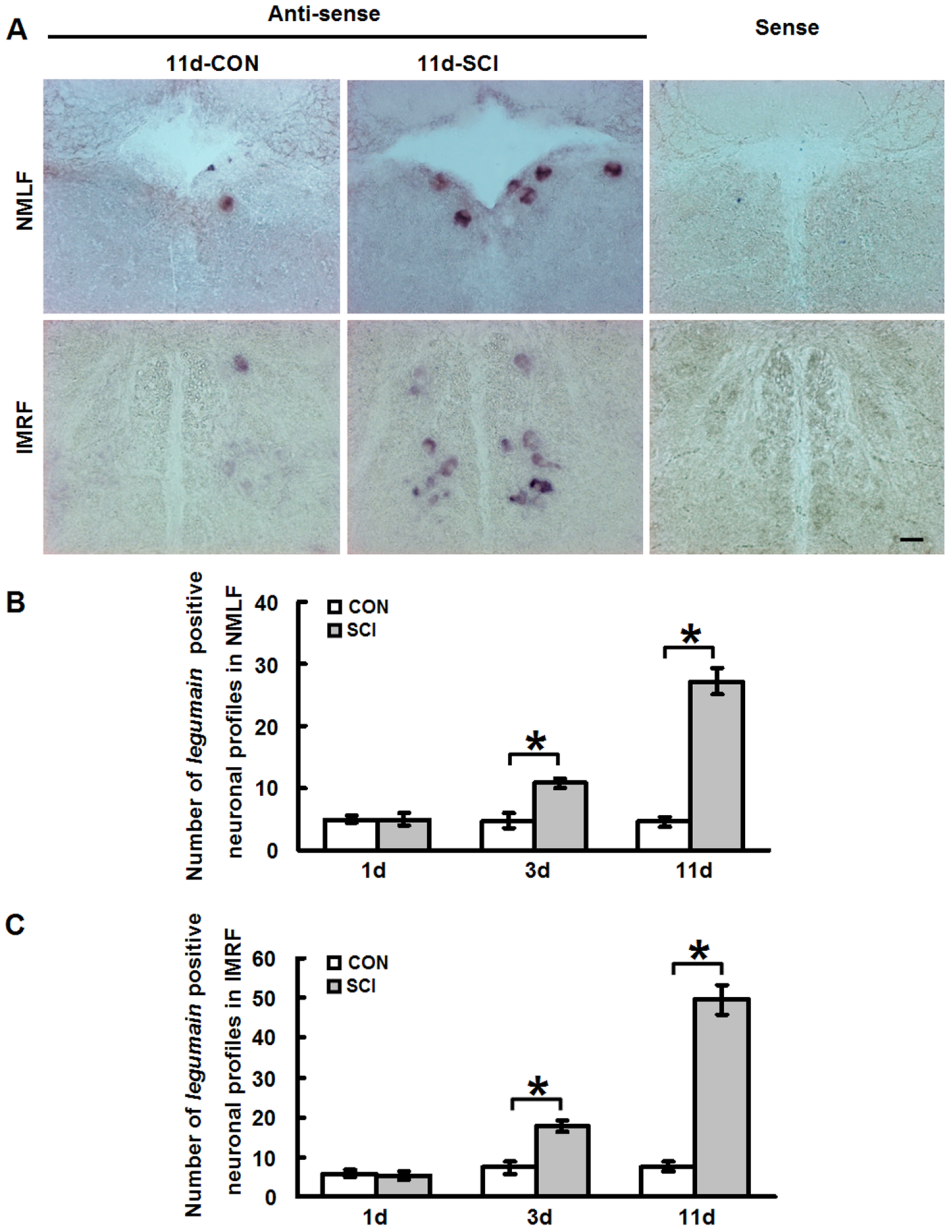
*Legumain* mRNA expression is upregulated in brainstem neurons during the axonal regrowth phase after SCI. (A) *In situ* hybridization was performed to study the expression of *legumain* in the NMLF and IMRF. Representative images depict *legumain*-positive cells in the NMLF and IMRF 11 days after SCI. More positive cells for *legumain* mRNA are observed in the NMLF and IMRF after SCI when compared with sham-injured control. With the sense control probe, no signal is observed. (B, C) Quantification shows that *legumain* mRNA expression is slightly upregulated in the NMLF and IMRF at 3 days and highly upregulated at 11 days after SCI. The expression patterns of *legumain* in the NMLF and IMRF are similar. Dorsal is up. NMLF, *n* = 6 fish; IMRF, *n* = 3 fish. * *P*<0.05, two-tailed *t*-test; mean values ±SEM are shown. Scale bar, 50 µm.

**Figure 2:**
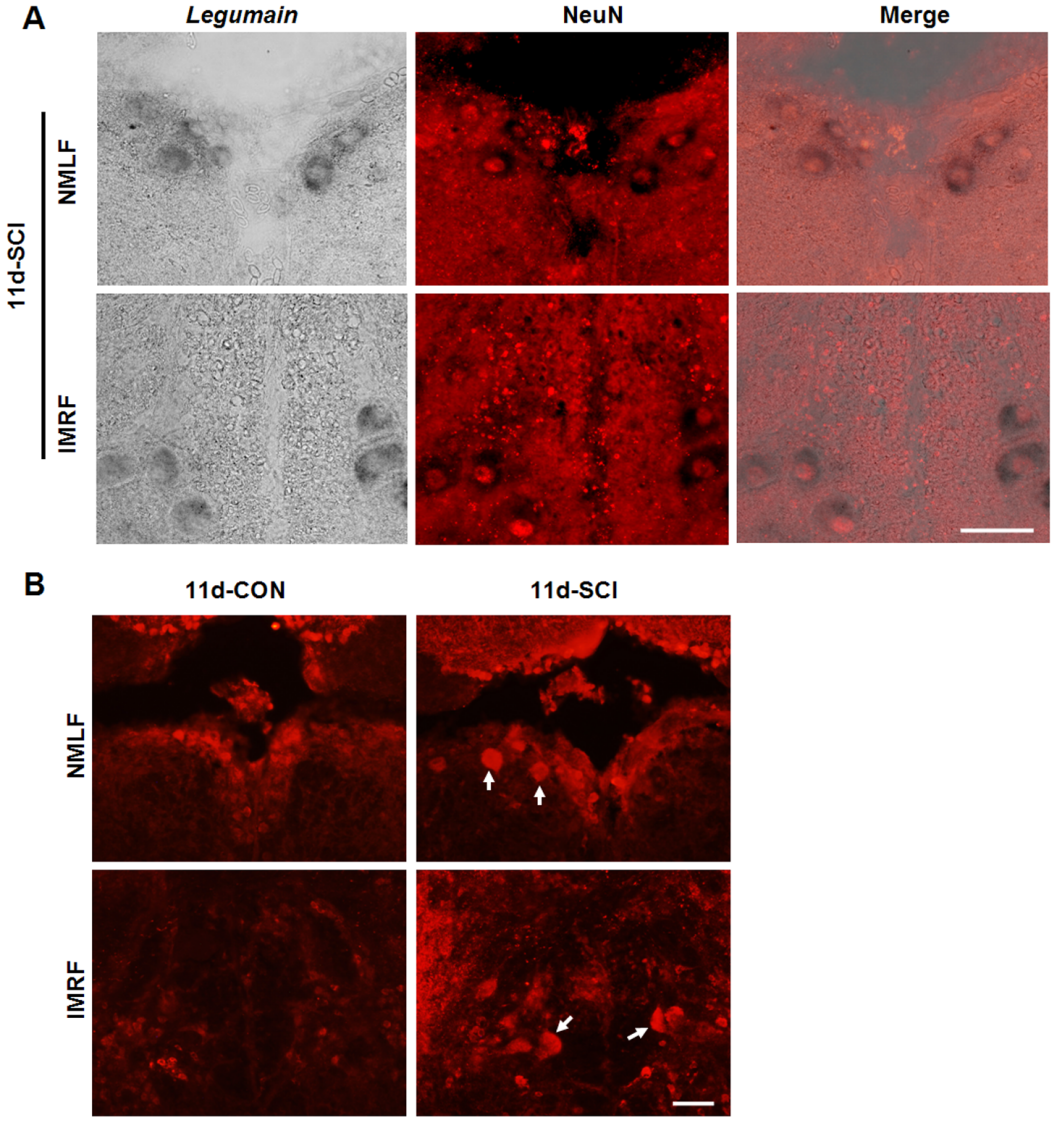
Upregulation of legumain expression in brainstem neurons at 11 days after SCI. (A) Double staining of *legumain* mRNA (*in situ* hybridization) and NeuN (immunohistochemistry) was performed to determine the identity of *legumain*-positive cells. The signal for NeuN locates in the nucleus and *legumain* mRNA locates in the neuronal cytoplasm in the NMLF and IMRF. (B) Immunohistochemistry for Legumain shows more Legumain positive neurons (arrows) in the NMLF and IMRF at 11 days after SCI when compared to the sham-injured control. Dorsal is up. A, *n* = 3 experiments; B, *n* = 3 fish. Scale bar, 50 µm.

To gain insights into the molecular mechanisms underlying successful spinal cord regeneration after SCI in adult zebrafish, we had performed microarray analysis to detect gene expression profile changes after SCI, when compared to sham-injured fish [2]. For the microarray analysis, RNA samples were prepared from tissue microdissected from the anatomically well defined NMLF nucleus. This analysis showed that *legumain* mRNA expression does not change during early phases after axotomy, i.e. 4 h (1.038±0.067 (SCI) versus 1.000±0.118 (CON), two-tailed *t*-test, *P*>0.05) and 12 h (0.954±0.120 (SCI) versus 1.000±0.089 (CON), two-tailed *t*-test, *P*>0.05) when compared to sham-injured fish, which were generally taken as controls. However, a significant upregulation of *legumain* mRNA expression was observed at 11 days (1.745±0.171 (SCI) versus 1.000±0.089 (CON), two-tailed *t*-test, *P*<0.05), a time point when tissue remodeling begins to lead to locomotor improvement. The expression of *legumain* mRNA in individual neurons in the NMLF and intermediate reticular formation (IMRF), another nucleus capable of innate regenerating lesioned axons, was studied by *in situ* hybridization. With the sense probe, no significant signal was observed when staining was performed in parallel using the antisense probe (Fig. 1A). Positive signal for *legumain* mRNA was observed in the NMLF neurons, which can easily be identified by their location and large cell body size (>13 µm in diameter) (Figs. 1A, 2A). The neuronal cell identity of these cells expressing *legumain* mRNA in the NMLF and IMRF was further validated by double labeling of *legumain* mRNA (*in situ* hybridization) and the neuronal marker NeuN (immunohistochemistry) (Fig. 2A). Consistent with our microarray analysis, the number of *legumain*-positive neuronal profiles was considerably increased in the NMLF at 11 days after SCI (Fig.1A, B). In the IMRF, upregulation of *legumain* mRNA expression was identified by increased numbers of positive neurons 11 days after SCI (Fig. 1A, C). Upregulation of *legumain* mRNA in these regenerative nuclei after SCI suggests that legumain contributes to successful regeneration.

In addition to investigating the nuclei with neurons capable of axonal regrowth, expression of *legumain* mRNA was also examined in neurons with no or limited capacity for innate regeneration, such as Mauthner neurons [20]. Unlike the findings in the NMLF or IMRF, no significant signal was observed in Mauthner neurons in sections from fish with or without SCI (data not shown). This indicates that upregulation of *legumain* mRNA is characteristic of neurons with regenerative capacity.

Microarray analysis showed no change for *legumain* mRNA expression at 4 h and 12 h after SCI in the NMLF. At 1 day after SCI, no change in *legumain* expression was observed in the NMLF (Fig. 1B) or IMRF (Fig. 1C). However, a slight but significant increase in *legumain* mRNA levels was observed at 3 days after SCI in both the NMLF (Fig. 1B) and IMRF (Fig. 1C), suggesting that upregulation of *legumain* expression as measured by *in situ* hybridization occurs at an early time point, but not immediately after axotomy. Upregulation of Legumain expression in the NMLF and IMRF was also seen at the protein level by immunohistochemistry using an antibody against human Legumain, which detects zebrafish Legumain (Fig. 2B). This upregulation of legumain expression by regenerative supraspinal neurons during axon regrowth/sprouting suggests that legumain contributes to regeneration after injury.

### *Legumain* expression is upregulated in the caudal spinal cord after SCI

**Figure 3:**
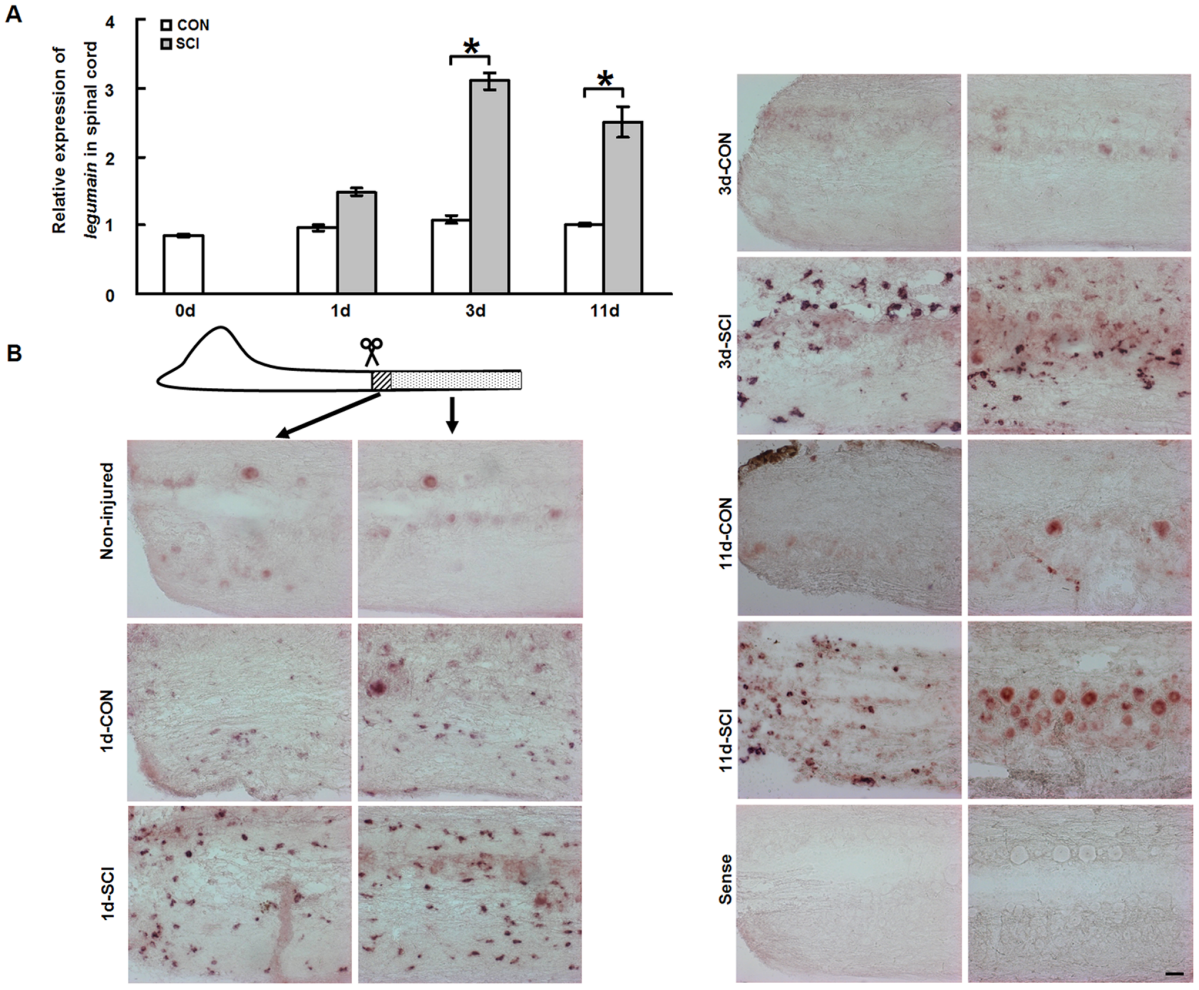
*Legumain* mRNA expression is upregulated in the caudal spinal cord after SCI. (A) Quantitative real-time PCR (qPCR) shows that *legumain* mRNA expression increases from 3 days post-injury and remains upregulated at 11 days after SCI. (B) *In situ* hybridization for *legumain* mRNA in the caudal spinal cord shows different expression patterns in the lesion site (area with stripes) and the caudal part of spinal cord (area with dots). In the non-injured spinal cord, *legumain* mRNA is only observed in neurons. In the lesion site, *legumain* mRNA is observed in many small cells at all time points tested after SCI. In the spinal cord caudal to the lesion site, *legumain* mRNA is detectable in small cells in both the sham-injured and SCI groups at 1 day, with a stronger signal for *legumain* mRNA after SCI compared to the sham injury group. At 3 days, while the signal for *legumain* mRNA in small cells is not detectable anymore in the sham-injured group, strong positive signal for *legumain* mRNA expression is observed in small cells in the SCI group. At 11 days, more positive neurons are seen after SCI compared to the sham-injury group. No signal is detectable with the sense probe. Rostral is left and caudal is right. A, *n* = 3 experiments; B, *n* = 4 fish for each group. * *P*<0.05, two-way ANOVA with Tukey's *post hoc* test; mean values ±SEM are shown. Scale bar, 50 µm.

In addition to brainstem neurons which are capable to regrow their axons after SCI, the caudal part spinal cord is another key factor for successful regeneration [19], [21], [29]–[33]. It is hypothesized that the caudal spinal cord, into which regenerating axons project, should be permissive for axonal regeneration, and that the cellular rearrangements in the caudal spinal cord also contribute essentially to regeneration. Levels of *legumain* mRNA in the caudal spinal cord were studied by qPCR at 1 day, 3 days and 11 days after SCI, in parallel with cells from the brainstem. Levels of *legumain* mRNA in the caudal spinal cord were not changed 1 day after SCI (Fig. 3A), but considerably increased at 3 days (Fig. 3A). Upregulation was also seen at 11 days after SCI (Fig. 3A), although less so than at 3 days after SCI.

To investigate the expression pattern of *legumain* in the caudal part of the injured spinal cord, *in situ* hybridization was performed. Two regions of the spinal cord were investigated: the tissue immediately caudal to the lesion site (approximately 50–80 µm in length) (Fig. 3B) and the remaining caudal spinal cord tissue (Fig. 3B). The reason for this analysis was that the two regions showed different *legumain* expression patterns by *in situ* hybridization after SCI. In the sham-injured control group, the two spinal cord regions showed a similar expression pattern of *legumain* (Fig. 3B).

In the non-injured spinal cord, *in situ* hybridization signals were mainly observed in a few neurons with large round cell bodies (>13 µm in diameter) in the gray matter. These cells were identified as neurons by double immunolabeling with the neuronal marker NeuN (see below). When compared to non-injured fish, positive signals in the 1 day sham-injured control spinal cord was also seen in many small cells with irregular shapes (identified as macrophages/microglia by double immunolabeling with 4C4 antibody, see below) localized in both gray and white matter along the entire length of the caudal spinal cord, indicating that sham-injury induces *legumain* expression. In comparison to the sham-injured group, more intense signals in these small cells were observed in the spinal cord caudal to the lesion site after SCI (Fig. 3B, 1d-CON, 100±8.55%; 1d-SCI, 186.11±6.24 (normalized to 1d-CON), *n* = 3 fish, two-tailed *t*-test, *P* = 0.001). No difference in the numbers of these small cells was found between the sham-injured and SCI groups (data not shown), indicating that the increased expression of *legumain* 1 day after SCI is due to increased expression intensity by the small cells. At the lesion site, at 1 day, 3 days and 11 days after SCI, many intensely *legumain* positive small cells were observed (Fig. 3B).

Interestingly, the *in situ* hybridization signal for *legumain* in the small cells in 3 days sham-injured control group was no longer detectable, and a positive signal was only seen in neurons as seen in the non-injured spinal cord. However, many small cells with strong signal for *legumain* were observed at 3 days after SCI. Furthermore, when compared to the signal at 1 day after SCI, more small cells were found in the spinal cord caudal to the injury site at 3 days after SCI (Fig. 3B, 1d-SCI, 100±5.02%; 3d-SCI, 149.42±12.03% (normalized to 1d-SCI), *n* = 3 fish, two-tailed *t*-test, *P* = 0.02), also with a more staining intense signal (Fig. 3B, 1d-SCI, 100±3.354%; 3d-SCI, 124.71±1.16% (normalized to 1d-SCI), *n* = 3 fish, two-tailed *t*-test, *P* = 0.002). Similar to the findings at 1 day after SCI, no significant differences of *legumain* expression in neurons were found between the sham-injured and SCI groups at 3 days. Thus, increased expression of *legumain* at 3 days after SCI is due to both the increased number of positive cells and expression intensity of *legumain* in the small cells compared to 1 day after SCI.

The expression pattern of *legumain* in the sham-injured group at 11 days was similar to that of the 3 day sham-injured group, i.e. only neurons with a large cell body (>13 µm in diameter) express *legumain* under these conditions. Interestingly, at 11 days after SCI, *legumain* in the small cells in the spinal cord caudal to the lesion site was no longer detectable, while more neurons showed upregulation of *legumain* expression after SCI when compared to the sham-injured control group (Fig. 3B, 11d-CON, 100±5.25%; 11d-SCI, 261.44±15.26% (normalized to 11d-CON), *n* = 3 fish, two-tailed *t*-test, *P* = 0.0006).

The combined observations indicate that upregulation of *legumain* expression in the caudal spinal cord is due to its expression by small cells in the lesion site at all time points tested. At 1 day and 3 days after SCI, upregulation of *legumain* is also due to increased expression by small cells in the spinal cord caudal to the lesion site. At 11 days after SCI, neurons contribute to this upregulation.

### Identification of legumain positive cells in the spinal cord

**Figure 4:**
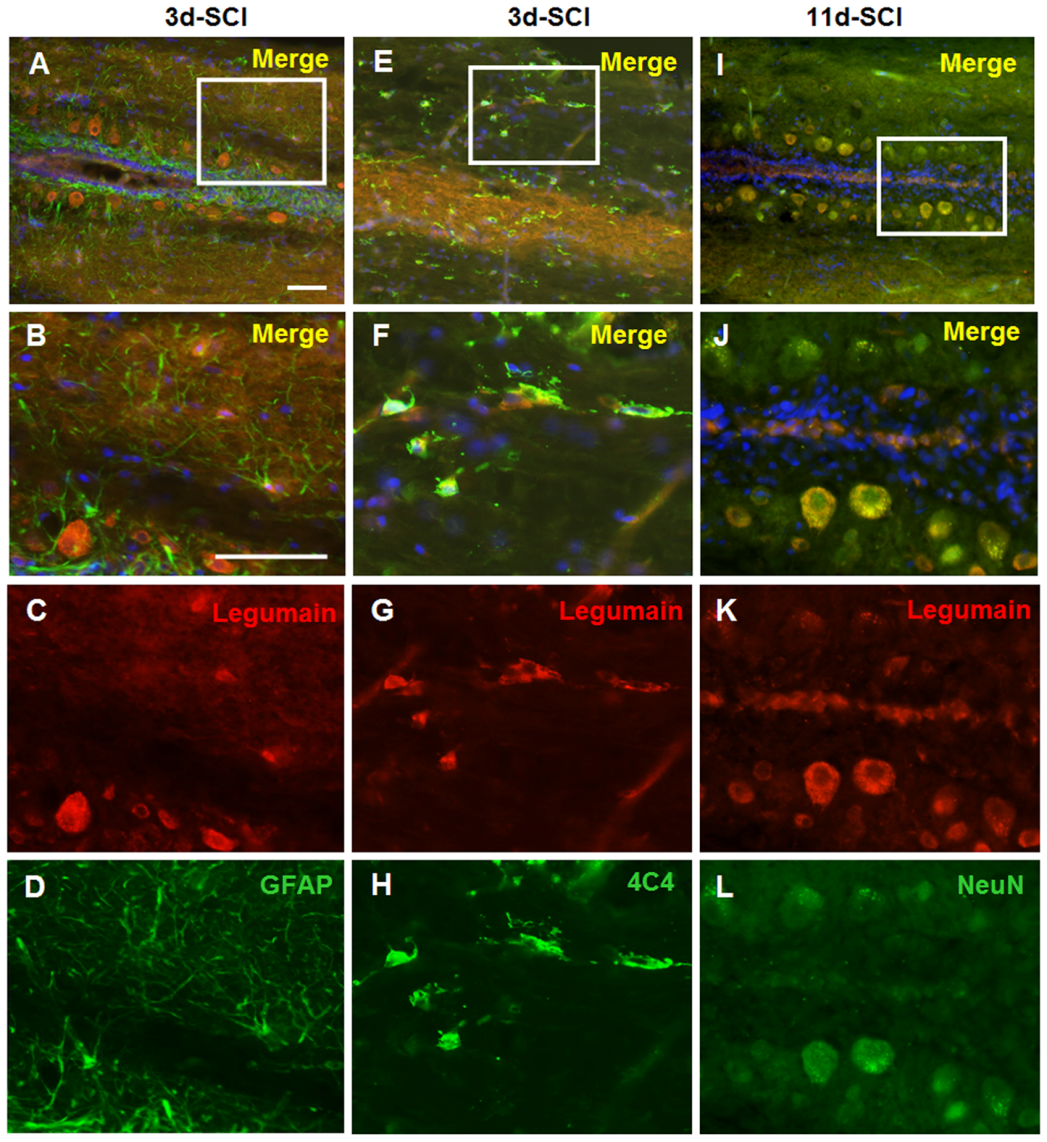
Neurons and macrophages/microglia express Legumain in the caudal spinal cord after SCI. Spinal cord sections from fish 3 days after SCI were used for double staining of Legumain and GFAP (A–D) or 4C4 (E–H). Spinal cord sections from fish 11 days after SCI were used for double staining of Legumain and NeuN (I–L). B, F, and J are the magnifications of A, E and I, respectively. No co-localization of Legumain and GFAP is observed (A–D). Double immunostaining of Legumain with 4C4 antibody identifies the positive small cells as macrophages/microglia (E–H). Double staining with NeuN shows that neurons express Legumain at 11 days after SCI (I–L). *n* = 3 experiments. Scale bar, 50 µm.

Cells expressing legumain in the spinal cord caudal to the lesion site were identified by immunohistochemistry using NeuN as a marker for neurons and GFAP for astrocytes. Macrophages/microglia were identified by antibody 4C4 [34]–[35]. Since highest expression of *legumain* by small cells was observed 3 days after SCI (Fig. 3B), double immunostaining of GFAP and 4C4 with Legumain was performed in sections taken from spinal cord caudal to the lesion site 3 days after SCI. Similarly, double immunostaining of NeuN with Legumain was performed using sections from fish 11 days after SCI, showing colocalization of NeuN with Legumain (Fig. 4 I–L). No colocalization GFAP and Legumain was observed (Fig. 4 A–D). In the non-injured spinal cord, only very few cells were positive for 4C4, which are negative for Legumain. Three days after SCI, almost all 4C4 positive cells expressed Legumain in the spinal cord caudal to the lesion site (Fig. 4 E–H). In the lesion site, Legumain expressing cells were also positive for 4C4 (data not shown). Interestingly, at 11 days after SCI, 4C4 immunopositive macrophages/microglia did not express detectable levels of Legumain (data not shown), demonstrating down-regulated expression of legumain in macrophages/microglia to basal levels as seen in the non-injured spinal cord. These observations suggest that expression of legumain in macrophages/microglia is limited to their early activation stages.

### Legumain is essential for spinal cord regeneration in adult zebrafish

**Figure 5:**
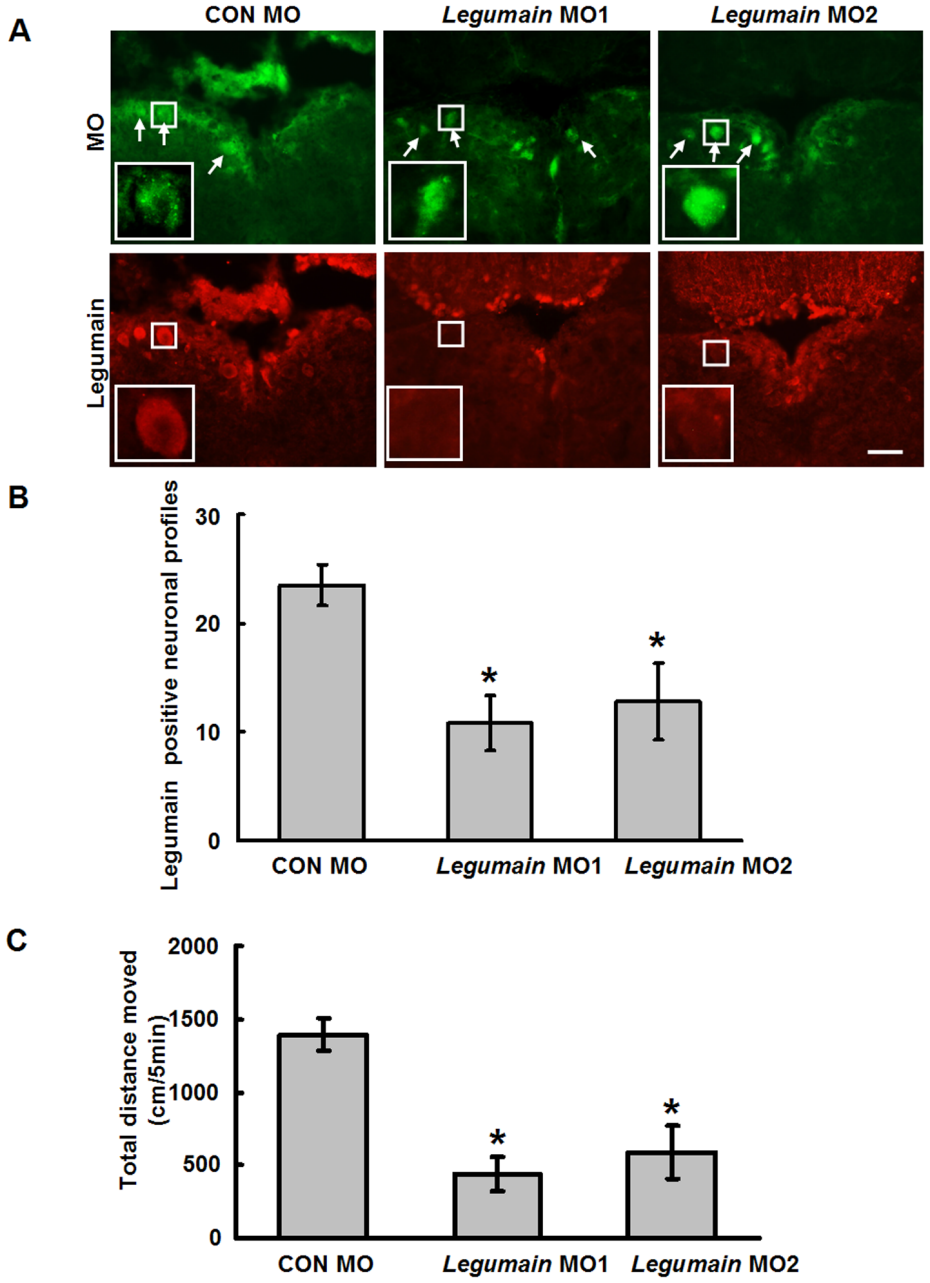
*Legumain* MO treatment inhibits locomotor recovery after SCI. (A, B) *Legumain* MO1 and MO2 knockdown Legumain protein expression as tested at 11 days after SCI. *Legumain* MO1 and *legumain* MO2 reduce the numbers of Legumain expressing neurons. Magnifications of the squared area are shown. (C) Total distance moved by fish treated with standard control (CON) MO, *legumain* MO1, or *legumain* MO2 was measured at 6 weeks after MO treatment. *Legumain* MO1 (*n* = 14 fish) or *legumain* MO2 (*n* = 11 fish) treatments reduce the total distance moved when compared with CON MO treatment (*n* = 11 fish). Dorsal is up. A, *n* = 3 experiments. * *P*<0.05, one-way ANOVA with Tukey's *post hoc* test; mean values ±SEM are shown. Scale bar, 50 µm.

As legumain expression is upregulated after SCI, we used anti-sense morpholinos (MOs) to knockdown expression of Legumain to investigate whether this molecule is essential in spinal cord regeneration. Two different MOs used here were labeled with fluorescein, allowing identification of the cells that had taken up the MOs. To validate the knockdown of Legumain at the protein level, immunohistochemistry was performed on brainstem sections from animals treated with standard control and legumain MOs 11 days after SCI. As shown in Figs. 1, 2 and 5, expression of Legumain in NMLF neurons was strongly increased after SCI. Application of *legumain* MO1 or MO2 (Fig. 5 A, B) dramatically reduced the numbers of Legumain protein expressing neurons in the NMLF compared to standard control MO treatment (Fig. 5 A, B). In the *legumain* anti-sense MO treated groups, neurons positive for fluorescein were not positive for Legumain by immunohistochemistry, while in the control MO treated group, neurons positive for MO also were positive for Legumain.

The locomotor recovery after complete spinal cord transection in adult zebrafish is measured by their free swimming ability at 6 weeks after SCI. The ability of injured fish to swim reaches maximal levels at 6 weeks after SCI with no additional improvement 10 weeks after SCI [19]. Locomotor recovery was quantified in terms of total distance moved by undisturbed fish during 5 min at 6 weeks after SCI. Fish treated with *legumain* MO1 or MO2 showed impaired recovery compared to fish treated with standard control MO: the total distances moved by fish treated with *legumain* MO1 (Fig. 5C) or *legumain* MO2 (Fig. 5C) were highly reduced relative to standard control MO treated fish, indicating that legumain contributes to functional recovery.

**Figure 6:**
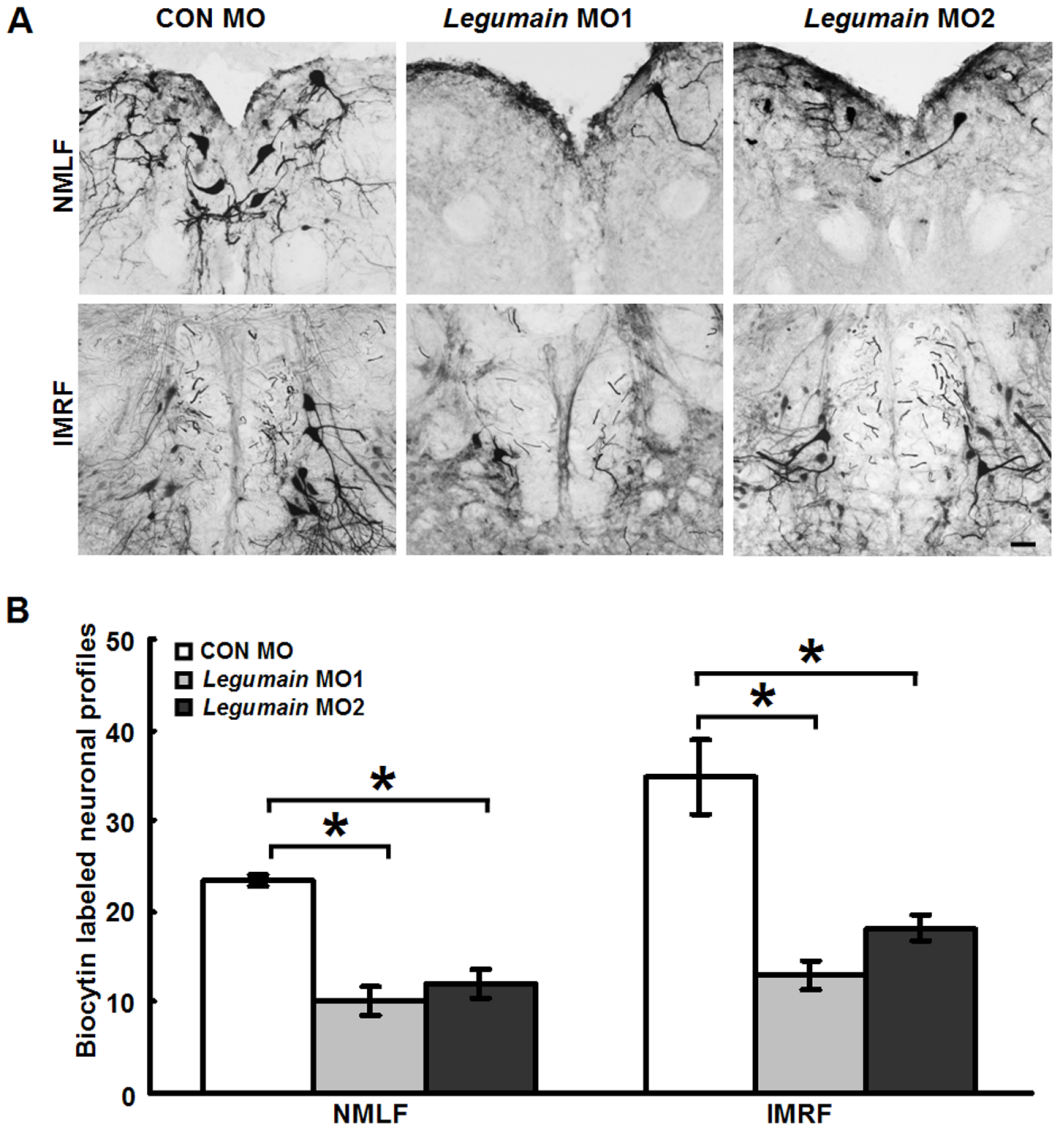
*Legumain* MOs inhibit axonal regrowth after SCI. (A) Representative images of neurons retrogradely labeled in the NMLF and IMRF 6 weeks after SCI. (B) Quantification of biocytin-labeled neuronal profiles in fish that had received *legumain* MO1 (*n* = 7 fish), *legumain* MO2 (*n* = 6 fish) or CON MO (*n* = 6 fish). *Legumain* MO1 and MO2 treatments reduce the numbers of biocytin-labeled neuronal profiles in the NMLF and IMRF when compared with CON MO treatment. Dorsal is up. * *P*<0.05, one-way ANOVA with Tukey's *post hoc* test; mean values ±SEM are shown. Scale bar, 50 µm.

To investigate the effect of *legumain* MOs on axonal regrowth after SCI, retrograde tracing of brainstem neurons was performed after analysis of locomotor recovery of the fish. The tracer, biocytin, was applied 3.5 mm caudal to the first lesion site. Fish treated with *legumain* anti-sense MOs showed reduced numbers of biocytin labeled neurons compared to control MO treated fish. The numbers of retrogradely labeled neurons were reduced in *legumain* anti-sense MO1 or MO2 treated fish in comparison to control MO treated fish in the NMLF or the IMRF (Fig. 6A, B). Since the *legumain* MOs were tagged with fluorescein, we were able to detect their presence 6 weeks after application as reported [2], [19]. As the standard control MO does not affect cell viability [19], we compared the numbers of fluorescein-positive cell profiles from *legumain* MO1 or MO2-treated fish with those from control MO-treated fish. No difference was found in the numbers of fluorescein-positive cells in the NMLF between control and experimental animals (data not shown), indicating that neurons are not affected in their survival by MO treatment. Altogether, observations showed reduced locomotor recovery and reduced numbers of retrogradely labeled NMLF neurons in animals treated with *legumain* MOs, indicating that legumain contributes to locomotor recovery and axonal regrowth after SCI in adult zebrafish.

## Discussion

In this study, we have identified legumain as an essential component for successful spinal cord regeneration after complete spinal cord transection in adult zebrafish. We found that legumain is one of the upregulated genes in the NMLF during axon regeneration by microarray analysis, *in situ* hybridization and immunohistochemistry. Moreover, levels of *legumain* mRNA are also increased in the caudal part spinal cord, which provides a permissive environment for regeneration. This upregulation of legumain expression has a biological function, since knockdown of Legumain expression strongly impaired locomotor recovery and axonal regrowth of brainstem neurons.

**Figure 7:**
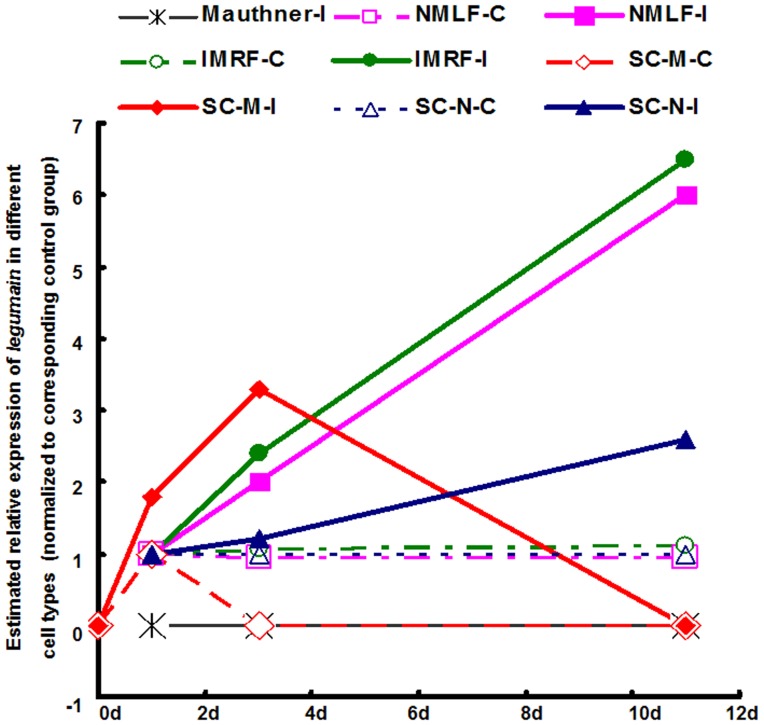
Summary scheme of *legumain* expression patterns in distinct cell types in brain and spinal cord after SCI. No signal for *legumain* expression was set as 0. When signal for *legumain* is detected in sham-injured control at 1 day post-injury, the value for *legumain* expression in 1 day sham-injured control is set as 1 and the relative expression in all other groups is normalized to the 1 day sham-injured control. The expression of *legumain* in brainstem neurons (NMLF-I, IMRF-I) is increased sharply after 1 day post-SCI. *Legumain* in spinal cord neurons (SC-N-I) is increased only at 11 days post-SCI. In the sham-injured control group, *legumain* expression in macrophages/microglia in the caudal spinal cord (SC-M-C) is detectable at 1 day and no more detectable thereafter. In the SCI group (SC-M-I), *legumain* expression in macrophages/microglia is increased 1 day, reaches a peak at 3 days and disappears 11 days after SCI. Abbreviations: Mauthner-I, Mauthner neurons of the SCI group; NMLF-C, NMLF neurons of the sham-injured control group; NMLF-I, NMLF neurons of the SCI group; IMRF-C, IMRF neurons of the sham-injured control group; IMRF-I, IMRF neurons of the SCI group; SC-M-C, macrophages/microglia in the caudal spinal cord of the sham-injured group; SC-M-I, macrophages/microglia in caudal spinal cord of the SCI group; SC-N-C, neurons in caudal spinal cord of the sham-injured group; SC-N-I, neurons in caudal spinal cord of the SCI group.

Microarray analysis had shown that there is no change of *legumain* mRNA expression during the early injury response period, i.e. 4 and 12 hours, suggesting that legumain is not involved in early responses after injury in neurons capable of axonal regeneration. At 3 days after SCI, upregulation of *legumain* expression becomes detectable and is very prominent at 11 days after SCI (Fig. 7), a time point of active axonal regrowth. Similar to our present results, upregulation of legumain expression occurs also at later phases after injury in retinal ganglion cells [36], optic nerve [37] and heart [38], suggesting that different organs use similar molecular mechanisms in regeneration. Similarly, upregulation of legumain is observed not only in the NMLF neurons, but also the IMRF neurons, indicating similar molecular mechanisms in neurons capable of axonal regrowth. Mauthner cells with no or limited capacity for regeneration, do not express *legumain* neither in sham-injured nor spinal cord injured fish at any time point after SCI, suggesting that upregulation of legumain expression is specifically associated with neurons capable of axonal regrowth.

In addition to the upregulation of *legumain* in brainstem neurons capable of axonal regeneration after SCI, cells in the spinal cord caudal to the lesion site also express higher levels of *legumain* after injury at all time points tested, with increased *legumain* expression reaching the highest level at 3 days after SCI. At 1 day and 3 days after SCI (Fig. 7), *legumain* expression is distinctly upregulated in macrophages/microglia, not only in the lesion site, but also caudal to the lesion site. Interestingly, however, *legumain* expression in macrophages/microglia is no more detectable at 11 days after SCI when neurons in spinal cord upregulate *legumain* expression (Fig. 7). Surprisingly, macrophages/microglia cells also express *legumain* in 1 day sham-injured control fish, in contrast to absence of *legumain* expression in these cells in non-injured fish. Of note, *legumain* expression in macrophages/microglia in sham-injured spinal cord is no more detectable at 3 days (Fig. 7), which differs from conditions of SCI under which *legumain* expression is upregulated at this time point. Insights into the molecular mechanisms underlying the transient expression after sham-injury are difficult to explain at present, but it is conceivable that de-afferentation of muscle innervation induced by cutting muscles at the thoracic level could activate macrophages/microglia. It should be emphasized that this transient *legumain* expression after sham-injury is weak in comparison to the reaction after SCI, but shows the sensitivity of macrophages/microglia to relatively mild interventions with physiological homeostasis. Similarly, legumain is detectable in mammalian macrophages [39]–[43] where its lysosomal proteolytic activity contributes to protein degradation and antigen presentation. However, the detailed molecular mechanisms of legumain's functions in macrophages/microglia have remained unclear. To the best of our knowledge, our study is the first to show that legumain expression is limited to a specifically transient stage of macrophages/microglia activation after tissue injury. Thus, legumain may become a useful early activation marker for macrophages/microglia.

The expression of legumain in macrophages/microglia raises the question as to the involvement of the immune system in zebrafish regeneration. In a recent study, acute inflammation initiates the regenerative response and is required for successful regeneration in the acutely injured adult zebrafish brain [44]. Similarly, application of anti-inflammatory glucocorticoid significantly reduces heart regenerative capacities in adult zebrafish [45]. Expression of legumain in macrophages/microglia in the early responses to injury in the spinal cord suggests that legumain expression might be supportive to recovery from trauma in zebrafish. Similarly, we found increased legumain expression at the lesion site of the mouse spinal cord at 7 days after SCI and this increase is attributable to accumulation of macrophages/microglia (unpublished observation). However, we did not observe upregulation of legumain in spinal motor neurons as we did in the zebrafish. In future experiments, we will test if increased expression of legumain in spinal cord and projection neurons can positively affect axon outgrowth after SCI in mammals.

After its discovery as a robust acidic cysteine endopeptidase, a considerable number of target proteins for legumain were described, such as extracellular matrix (ECM) component fibronectin [12]. The expression and functions of legumain in solid tumors has also been explored. In contrast to its low expression level in most normal tissues [9], [46], legumain is highly expressed in many solid tumors, being related to a more invasive and metastatic phenotype [9], [16]–[17], [47]. Tumor invasion and metastasis is a complex process involving interactions between the invasive cells and the ECM, where proteases are essential for tumor cell-mediated ECM proteolysis. Notably, legumain is present extracellularly in the tumor microenviroment and associated with matrix as well as cell surfaces [9], [11], [48]. The association of secreted legumain with the extracellular matrix has been repeatedly described [10]–[13], [16], [49], an observation which has instigated the design legumain-activated anti-cancer prodrugs [13]. Furthermore, legumain is able to activate the secreted inactive proenzyme of matrix metalloproteinase-2 [9], [50], the role of which in tumor cell-mediated ECM proteolysis and metastasis has been well established [51]. Similar to mechanisms in tumor metastasis, growth cones of regenerating axons degrade ECM molecules for motility and neurite extension, pointing to share similar mechanisms in proteolysis [52]. The extracellular activity of legumain is indicated in our study, where we could show that legumain is secreted by legumain positive cell types, such as microglia/macrophages and neurons (Figure S2). It is also possible that the upregulation of legumain in the caudal spinal cord after SCI may play a role in remodeling the extracellular environment to facilitate axon regeneration into the caudal spinal cord. It is noteworthy in this context that legumain is optimally active under acidic conditions, which are acutely generated in the spinal cord after injury and which are present in the microenvironment of tumors. Unfortunately, the investigation of the mechanisms underlying the functions of legumain with different cell types *in vitro* is hampered by the fact that cultured cells cannot be maintained under acidic conditions for time periods necessary for functional studies, thereby preventing the establishment of conditions that mirror those *in vivo* after injury.

It is noteworthy that some functions of legumain do not require its enzyme activity. The expression of legumain in the nucleus is regulated by nuclear calcium concentration and nuclear legumain is involved in cell proliferation, independent on its enzyme activity [7]. Also legumain's presence in human bone marrow is reported to inhibit osteoclast formation and bone resorption without its enzymatic activity [10]. Moreover, identification of legumain as a carboxypeptidase may allow novel insights into the functions of legumain under different physiological conditions [18]. In future experiments, we plan to examine the mechanism through which legumain appears to enhance recovery from CNS injury in zebrafish. Discovery of other target proteins for legumain and further biochemical studies may be helpful in elucidating mechanistic features of legumain function to develop potential therapies to promote CNS repair in mammals.

## Supporting Information

Figure S1 **Determination of the ability of legumain antibody to specifically detect Legumain in zebrafish as assayed by Western blot analysis.** N2a cells was used as positive control (lane 1) and zebrafish spinal cord was used for this analysis (lane 3). Protein marker was loaded in lane 2. The antibody detects two major bands at 50 kDa and 37 kDa with N2a cells, the inactive proenzyme form and active form of mouse legumain, respectively. A band with the same size at 50 kDa is detected with zebrafish spinal cord as for N2a cells. n = 3 experiments.
(TIF)

Figure S2 **Assays for detecting secretion of Legumain by different cell types** ***in vitro*** **.** The secreted Legumain is mainly presented by the inactive proenzyme form at 50 kDa (the bands with arrows) in Western blot analysis. (A) Cultured macrophages secrete Legumain into the culture medium (lane 2) compared to non-cultured fresh control medium (lane 1). (B) N2a neuroblastoma cells (lane 2) secrete Legumain into the culture medium compared to non-cultured control medium (lane 1). (C) Primary cultured E18 embryonic hippocampal neurons secrete Legumain into the medium as tested by ELISA compared to non-cultured control medium. * *P*<0.05, two-tailed *t*-test; mean values ±SEM are shown. n = 3 experiments.
(TIF)

## References

[1] Becker CG, Becker T (2008) Adult zebrafish as a model for successful central nervous system regeneration. Restor Neurol Neurosci 26: 71–80. 18820403

[2] Ma L, Yu YM, Guo Y, Hart RP, Schachner M (2012) Cysteine- and glycine-rich protein 1a is involved in spinal cord regeneration in adult zebrafish. Eur J Neurosci 35: 353–365. 22288476 10.1111/j.1460-9568.2011.07958.x PMC4442618

[3] Van Hove I, Lemmens K, Van de Velde S, Verslegers M, Moons L (2012) Matrix metalloproteinase-3 in the central nervous system: a look on the bright side. J Neurochem 123: 203–216. 22862420 10.1111/j.1471-4159.2012.07900.x

[4] Zhang H, Chang M, Hansen CN, Basso DM, Noble-Haeusslein LJ (2011) Role of matrix metalloproteinases and therapeutic benefits of their inhibition in spinal cord injury. Neurotherapeutics 8: 206–220. 21455784 10.1007/s13311-011-0038-0 PMC3077748

[5] Chen JM, Dando PM, Rawlings ND, Brown MA, Young NE, et al (1997) Cloning, isolation, and characterization of mammalian legumain, an asparaginyl endopeptidase. J Biol Chem 272: 8090–8098. 9065484 10.1074/jbc.272.12.8090

[6] Chen JM, Dando PM, Stevens RA, Fortunato M, Barrett AJ (1998) Cloning and expression of mouse legumain, a lysosomal endopeptidase. Biochem J 335 (Pt 1): 111–117. 10.1042/bj3350111 PMC1219758 9742219

[7] Andrade V, Guerra M, Jardim C, Melo F, Silva W, et al (2011) Nucleoplasmic calcium regulates cell proliferation through legumain. J Hepatol 55: 626–635. 21237226 10.1016/j.jhep.2010.12.022 PMC3158841

[8] Haugen MH, Johansen HT, Pettersen SJ, Solberg R, Brix K, et al (2013) Nuclear legumain activity in colorectal cancer. PLoS One 8: e52980. 23326369 10.1371/journal.pone.0052980 PMC3542341

[9] Liu C, Sun C, Huang H, Janda K, Edgington T (2003) Overexpression of legumain in tumors is significant for invasion/metastasis and a candidate enzymatic target for prodrug therapy. Cancer Res 63: 2957–2964. 12782603

[10] Choi SJ, Reddy SV, Devlin RD, Menaa C, Chung H, et al (1999) Identification of human asparaginyl endopeptidase (legumain) as an inhibitor of osteoclast formation and bone resorption. J Biol Chem 274: 27747–27753. 10488118 10.1074/jbc.274.39.27747

[11] Liu Y, Bajjuri KM, Liu C, Sinha SC (2012) Targeting cell surface alpha(v)beta(3) integrin increases therapeutic efficacies of a legumain protease-activated auristatin prodrug. Mol Pharm 9: 168–175. 22044266 10.1021/mp200434n PMC3277864

[12] Morita Y, Araki H, Sugimoto T, Takeuchi K, Yamane T, et al (2007) Legumain/asparaginyl endopeptidase controls extracellular matrix remodeling through the degradation of fibronectin in mouse renal proximal tubular cells. FEBS Lett 581: 1417–1424. 17350006 10.1016/j.febslet.2007.02.064

[13] Wu W, Luo Y, Sun C, Liu Y, Kuo P, et al (2006) Targeting cell-impermeable prodrug activation to tumor microenvironment eradicates multiple drug-resistant neoplasms. Cancer Res 66: 970–980. 16424032 10.1158/0008-5472.CAN-05-2591

[14] Manoury B, Hewitt EW, Morrice N, Dando PM, Barrett AJ, et al (1998) An asparaginyl endopeptidase processes a microbial antigen for class II MHC presentation. Nature 396: 695–699. 9872320 10.1038/25379

[15] Shirahama-Noda K, Yamamoto A, Sugihara K, Hashimoto N, Asano M, et al (2003) Biosynthetic processing of cathepsins and lysosomal degradation are abolished in asparaginyl endopeptidase-deficient mice. J Biol Chem 278: 33194–33199. 12775715 10.1074/jbc.M302742200

[16] Briggs JJ, Haugen MH, Johansen HT, Riker AI, Abrahamson M, et al (2010) Cystatin E/M suppresses legumain activity and invasion of human melanoma. BMC Cancer 10: 17. 20074384 10.1186/1471-2407-10-17 PMC2822816

[17] Ohno Y, Nakashima J, Izumi M, Ohori M, Hashimoto T, et al (2013) Association of legumain expression pattern with prostate cancer invasiveness and aggressiveness. World J Urol 31: 359–364. 23124822 10.1007/s00345-012-0977-z

[18] Dall E, Brandstetter H (2013) Mechanistic and structural studies on legumain explain its zymogenicity, distinct activation pathways, and regulation. Proc Natl Acad Sci U S A 110: 10940–10945. 23776206 10.1073/pnas.1300686110 PMC3703970

[19] Becker CG, Lieberoth BC, Morellini F, Feldner J, Becker T, et al (2004) L1.1 is involved in spinal cord regeneration in adult zebrafish. J Neurosci 24: 7837–7842. 15356195 10.1523/JNEUROSCI.2420-04.2004 PMC6729920

[20] Becker T, Wullimann MF, Becker CG, Bernhardt RR, Schachner M (1997) Axonal regrowth after spinal cord transection in adult zebrafish. J Comp Neurol 377: 577–595. 9007194 10.1002/(sici)1096-9861(19970127)377:4<577::aid-cne8>3.0.co;2-#

[21] Guo Y, Ma L, Cristofanilli M, Hart RP, Hao A, et al (2011) Transcription factor Sox11b is involved in spinal cord regeneration in adult zebrafish. Neuroscience 172: 329–341. 20951776 10.1016/j.neuroscience.2010.10.026 PMC3292217

[22] Yu YM, Cristofanilli M, Valiveti A, Ma L, Yoo M, et al (2011) The extracellular matrix glycoprotein tenascin-C promotes locomotor recovery after spinal cord injury in adult zebrafish. Neuroscience 183: 238–250. 21443931 10.1016/j.neuroscience.2011.03.043

[23] Yu YM, Gibbs KM, Davila J, Campbell N, Sung S, et al (2011) MicroRNA miR-133b is essential for functional recovery after spinal cord injury in adult zebrafish. Eur J Neurosci 33: 1587–1597. 21447094 10.1111/j.1460-9568.2011.07643.x PMC3100659

[24] Goff LA, Bowers J, Schwalm J, Howerton K, Getts RC, et al (2004) Evaluation of sense-strand mRNA amplification by comparative quantitative PCR. BMC Genomics 5: 76. 15469607 10.1186/1471-2164-5-76 PMC524485

[25] Becker T, Bernhardt RR, Reinhard E, Wullimann MF, Tongiorgi E, et al (1998) Readiness of zebrafish brain neurons to regenerate a spinal axon correlates with differential expression of specific cell recognition molecules. J Neurosci 18: 5789–5803. 9671667 10.1523/JNEUROSCI.18-15-05789.1998 PMC6793072

[26] Lieberoth BC, Becker CG, Becker T (2003) Double labeling of neurons by retrograde axonal tracing and non-radioactive in situ hybridization in the CNS of adult zebrafish. Methods Cell Sci 25: 65–70. 14739589 10.1023/B:MICS.0000006848.57869.4c

[27] Wullimann MF, B Rupp, and H Reichert (1996) Neuroanatomy of the Zebrafish Brain: A Topological Atlas. Basel: Birkhauser

[28] Ma L, Yin M, Wu X, Wu C, Yang S, et al (2006) Expression of trophinin and bystin identifies distinct cell types in the germinal zones of adult rat brain. Eur J Neurosci 23: 2265–2276. 16706835 10.1111/j.1460-9568.2006.04782.x

[29] Lin JF, Pan HC, Ma LP, Shen YQ, Schachner M (2012) The cell neural adhesion molecule contactin-2 (TAG-1) is beneficial for functional recovery after spinal cord injury in adult zebrafish. PLoS One 7: e52376. 23285014 10.1371/journal.pone.0052376 PMC3528781

[30] Pan HC, Lin JF, Ma LP, Shen YQ, Schachner M (2013) Major vault protein promotes locomotor recovery and regeneration after spinal cord injury in adult zebrafish. Eur J Neurosci 37: 203–211. 23106570 10.1111/ejn.12038

[31] Yu Y, Schachner M (2013) Syntenin-a promotes spinal cord regeneration following injury in adult zebrafish. Eur J Neurosci 38: 2280–2289. 23607754 10.1111/ejn.12222

[32] Dias TB, Yang YJ, Ogai K, Becker T, Becker CG (2012) Notch signaling controls generation of motor neurons in the lesioned spinal cord of adult zebrafish. J Neurosci 32: 3245–3252. 22378895 10.1523/JNEUROSCI.6398-11.2012 PMC6622036

[33] Reimer MM, Kuscha V, Wyatt C, Sorensen I, Frank RE, et al (2009) Sonic hedgehog is a polarized signal for motor neuron regeneration in adult zebrafish. J Neurosci 29: 15073–15082. 19955358 10.1523/JNEUROSCI.4748-09.2009 PMC2841428

[34] Baumgart EV, Barbosa JS, Bally-Cuif L, Gotz M, Ninkovic J (2012) Stab wound injury of the zebrafish telencephalon: a model for comparative analysis of reactive gliosis. Glia 60: 343–357. 22105794 10.1002/glia.22269

[35] Becker T, Becker CG (2001) Regenerating descending axons preferentially reroute to the gray matter in the presence of a general macrophage/microglial reaction caudal to a spinal transection in adult zebrafish. J Comp Neurol 433: 131–147. 11283955 10.1002/cne.1131

[36] Veldman MB, Bemben MA, Thompson RC, Goldman D (2007) Gene expression analysis of zebrafish retinal ganglion cells during optic nerve regeneration identifies KLF6a and KLF7a as important regulators of axon regeneration. Dev Biol 312: 596–612. 17949705 10.1016/j.ydbio.2007.09.019

[37] McCurley AT, Callard GV (2010) Time Course Analysis of Gene Expression Patterns in Zebrafish Eye During Optic Nerve Regeneration. J Exp Neurosci 2010: 17–33. 20740047 PMC2926816

[38] Lien CL, Schebesta M, Makino S, Weber GJ, Keating MT (2006) Gene expression analysis of zebrafish heart regeneration. PLoS Biol 4: e260. 16869712 10.1371/journal.pbio.0040260 PMC1523227

[39] Edgington LE, Verdoes M, Ortega A, Withana NP, Lee J, et al (2013) Functional imaging of legumain in cancer using a new quenched activity-based probe. J Am Chem Soc 135: 174–182. 23215039 10.1021/ja307083b PMC4429797

[40] Hashimoto S, Suzuki T, Dong HY, Yamazaki N, Matsushima K (1999) Serial analysis of gene expression in human monocytes and macrophages. Blood 94: 837–844. 10419873

[41] Lecaille F, Muno D, Kominami E, Ishidoh K (2004) Proteinases participating in the processing and activation of prolegumain in primary cultured rat macrophages. Biol Chem 385: 511–516. 15255183 10.1515/BC.2004.060

[42] Lin Y, Wei C, Liu Y, Qiu Y, Liu C, et al (2013) Selective ablation of tumor-associated macrophages suppresses metastasis and angiogenesis. Cancer Sci 104: 1217–1225. 23691974 10.1111/cas.12202 PMC3766435

[43] Luo Y, Zhou H, Krueger J, Kaplan C, Lee SH, et al (2006) Targeting tumor-associated macrophages as a novel strategy against breast cancer. J Clin Invest 116: 2132–2141. 16862213 10.1172/JCI27648 PMC1513049

[44] Kyritsis N, Kizil C, Zocher S, Kroehne V, Kaslin J, et al (2012) Acute inflammation initiates the regenerative response in the adult zebrafish brain. Science 338: 1353–1356. 23138980 10.1126/science.1228773

[45] Huang WC, Yang CC, Chen IH, Liu YM, Chang SJ, et al (2013) Treatment of Glucocorticoids Inhibited Early Immune Responses and Impaired Cardiac Repair in Adult Zebrafish. PLoS One 8: e66613. 23805247 10.1371/journal.pone.0066613 PMC3689762

[46] Yamane T, Takeuchi K, Yamamoto Y, Li YH, Fujiwara M, et al (2002) Legumain from bovine kidney: its purification, molecular cloning, immunohistochemical localization and degradation of annexin II and vitamin D-binding protein. Biochim Biophys Acta 1596: 108–120. 11983426 10.1016/s0167-4838(02)00209-1

[47] Wang L, Chen S, Zhang M, Li N, Chen Y, et al (2012) Legumain: a biomarker for diagnosis and prognosis of human ovarian cancer. J Cell Biochem 113: 2679–2686. 22441772 10.1002/jcb.24143

[48] Wu B, Yin J, Texier C, Roussel M, Tan KS (2010) Blastocystis legumain is localized on the cell surface, and specific inhibition of its activity implicates a pro-survival role for the enzyme. J Biol Chem 285: 1790–1798. 19915007 10.1074/jbc.M109.049064 PMC2804337

[49] Smith R, Johansen HT, Nilsen H, Haugen MH, Pettersen SJ, et al (2012) Intra- and extracellular regulation of activity and processing of legumain by cystatin E/M. Biochimie 94: 2590–2599. 22902879 10.1016/j.biochi.2012.07.026

[50] Chen JM, Fortunato M, Stevens RA, Barrett AJ (2001) Activation of progelatinase A by mammalian legumain, a recently discovered cysteine proteinase. Biol Chem 382: 777–783. 11517930 10.1515/BC.2001.093

[51] Deryugina EI, Quigley JP (2006) Matrix metalloproteinases and tumor metastasis. Cancer Metastasis Rev 25: 9–34. 16680569 10.1007/s10555-006-7886-9

[52] Zuo J, Ferguson TA, Hernandez YJ, Stetler-Stevenson WG, Muir D (1998) Neuronal matrix metalloproteinase-2 degrades and inactivates a neurite-inhibiting chondroitin sulfate proteoglycan. J Neurosci 18: 5203–5211. 9651203 10.1523/JNEUROSCI.18-14-05203.1998 PMC6793496

