# Supplementary figures and images for: The Asparaginyl Endopeptidase Legumain Is Essential for Functional Recovery after Spinal Cord Injury in Adult Zebrafish
Source: PLoS One. 2014 Apr 18;9(4):e95098. doi: 10.1371/journal.pone.0095098 (PMC3991597; doi:10.1371/journal.pone.0095098)

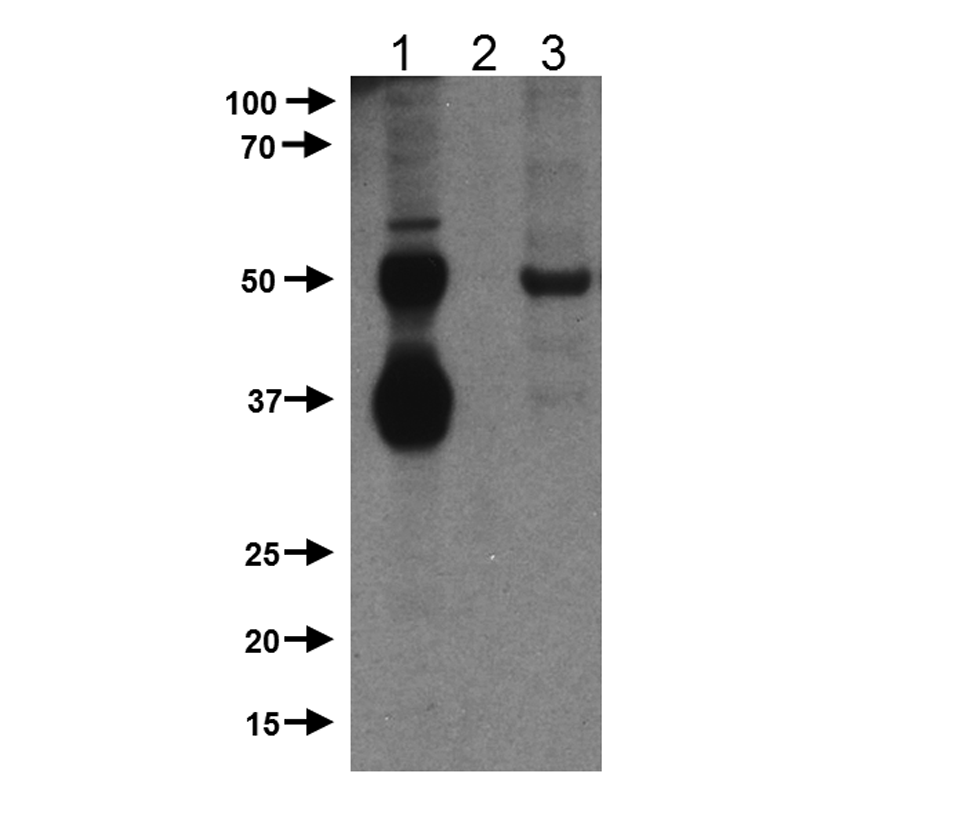

Supplement: Figure S1 — Determination of the ability of legumain antibody to specifically detect Legumain in zebrafish as assayed by Western blot analysis. N2a cells was used as positive control (lane 1) and zebrafish spinal cord was used for this analysis (lane 3). Protein marker was loaded in lane 2. The antibody detects two major bands at 50 kDa and 37 kDa with N2a cells, the inactive proenzyme form and active form of mouse legumain, respectively. A band with the same size at 50 kDa is detected with zebrafish spinal cord as for N2a cells. n = 3 experiments. (TIF) [file pone.0095098.s001.tif]

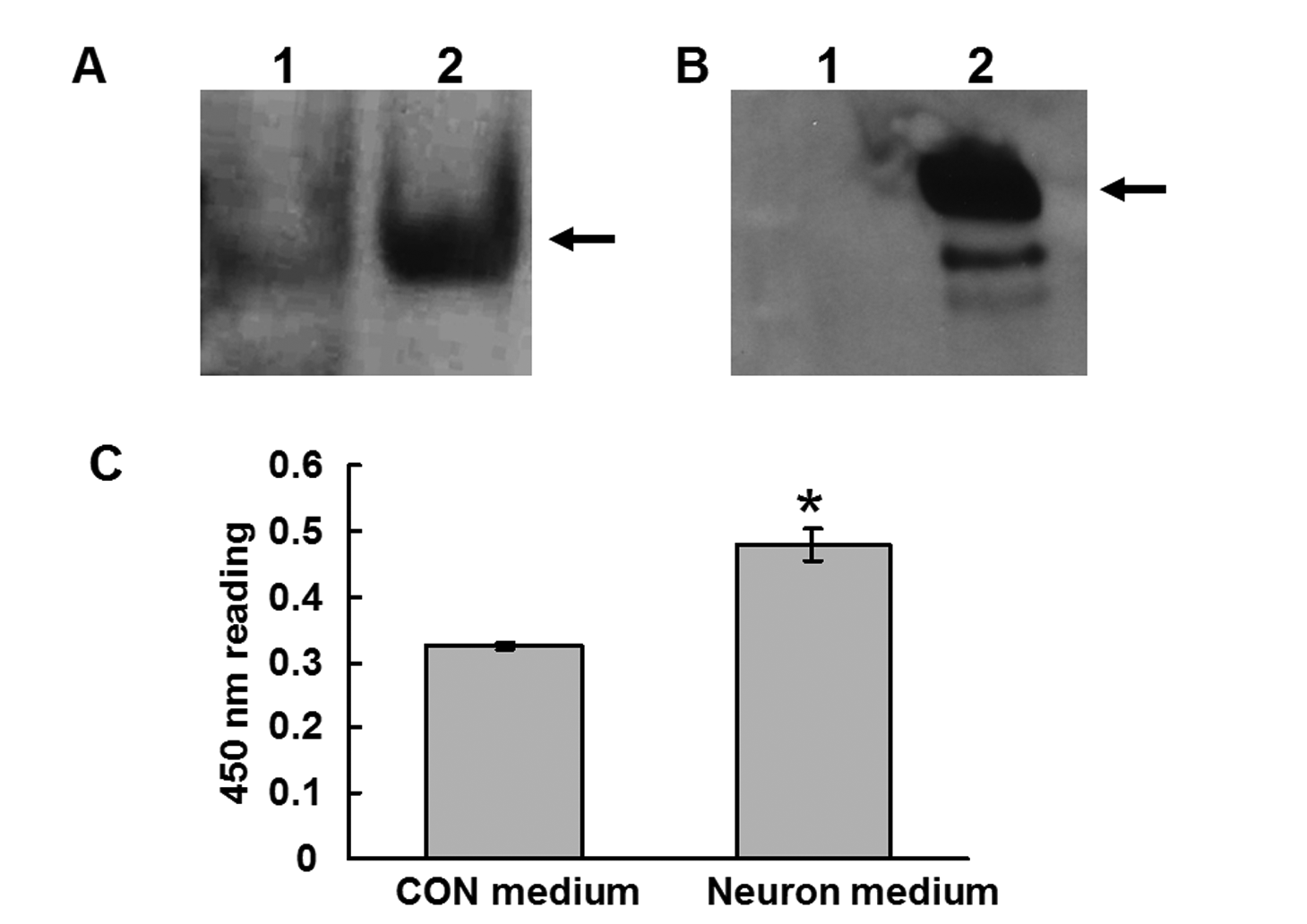

Supplement: Figure S2 — Assays for detecting secretion of Legumain by different cell types in vitro . The secreted Legumain is mainly presented by the inactive proenzyme form at 50 kDa (the bands with arrows) in Western blot analysis. (A) Cultured macrophages secrete Legumain into the culture medium (lane 2) compared to non-cultured fresh control medium (lane 1). (B) N2a neuroblastoma cells (lane 2) secrete Legumain into the culture medium compared to non-cultured control medium (lane 1). (C) Primary cultured E18 embryonic hippocampal neurons secrete Legumain into the medium as tested by ELISA compared to non-cultured control medium. * P<0.05, two-tailed t-test; mean values ±SEM are shown. n = 3 experiments. (TIF) [file pone.0095098.s002.tif]
